# Association Between Ultrasound Parameters and History of Ischemic or Hemorrhagic Stroke in Patients With Moyamoya Disease

**DOI:** 10.3389/fneur.2021.570843

**Published:** 2021-02-15

**Authors:** Shuai Zheng, Peicong Ge, Yi Li, Jingzhe Wang, Zhiyong Shi, Jinghan Zhang, Lei He, Linggang Cheng, Dong Zhang, Wen He

**Affiliations:** ^1^Department of Ultrasound, Beijing Tiantan Hospital, Capital Medical University, Beijing, China; ^2^Department of Neurosurgery, Beijing Tiantan Hospital, Capital Medical University, Beijing, China; ^3^China National Clinical Research Center for Neurological Diseases, Beijing, China; ^4^Center of Stroke, Beijing Institute for Brain Disorders, Beijing, China; ^5^Beijing Key Laboratory of Translational Medicine for Cerebrovascular Disease, Beijing, China; ^6^Beijing Translational Engineering Center for 3D Printer in Clinical Neuroscience, Beijing, China; ^7^Department of Ultrasound, Tianjin Medical University Cancer Institute and Hospital, Tianjin, China

**Keywords:** moyamoya disease, stroke, ultrasound, nomogram, extracranial internal carotid artery, posterior cerebral artery

## Abstract

**Objective:** To explore the association between ultrasound parameters and previous ischemic or hemorrhagic stroke in patients with moyamoya disease (MMD), and develop an ultrasound-based nomogram to identify stroke in patients with MMD.

**Methods:** We prospectively enrolled 52 consecutive patients (92 hemispheres) with MMD at the Beijing Tiantan Hospital. Thirty-six patients (65 hemispheres) were assigned to the training dataset from September 2019 to February 2020, and 16 patients (27 hemispheres) were assigned to the validation dataset from March 2020 to July 2020. Multivariate logistic regression analysis was applied to identify ultrasound parameters associated with previous history of ipsilateral stroke in patients with MMD, and a nomogram was subsequently constructed to identify stroke in patients with MMD. The performance of the nomogram was evaluated with respect to discrimination, calibration, and clinical usefulness.

**Results:** Multivariate analysis indicated that the flow volume (FV) of the extracranial internal carotid artery (EICA) and the peak systolic velocity (PSV) of the posterior cerebral artery (PCA) were independently associated with ipsilateral stroke in patients with MMD, a nomogram incorporating these two parameters was constructed to identify stroke patients. The area under the receiver operating characteristic (AUROC) curves was 0.776 (95% CI, 0.656–0.870) in the training dataset and 0.753 (95% CI, 0.550–0.897) in the validation dataset suggested that the model had good discrimination ability. The calibration plot showed good agreement in both the two datasets. The decision curve analysis (DCA) revealed that the nomogram was clinically useful.

**Conclusions:** Ultrasound parameters of EICA and PCA are independently associated with history of previous ipsilateral ischemic or hemorrhagic stroke in patients with MMD. The present ultrasound-based nomogram might provide information to identify MMD patients with high risk of stroke. Future long-term follow-up studies are needed to prove the predictive value in other independent cohorts.

**Clinical Trial Registration:** http://www.chictr.org.cn/index.aspx. Unique Identifier: ChiCTR1900026075.

## Introduction

Moyamoya disease (MMD), which is a rare disease with unknown etiology, predisposes some patients to stroke. It is characterized by progressive stenosis of the terminal portions of the internal carotid arteries and their proximal branches. Reduced blood flow in the main vessels of the intracranial anterior circulation leads to compensatory development of abnormal vascular network (1, 2). Leptomeningeal collateral vessels from the posterior cerebral artery (PCA) are regarded as the main collateral vessels in patients with MMD, and transdural collaterals from the branches of external carotid artery (ECA), including middle meningeal artery, maxillary artery (MA), superficial temporal artery (STA) can also compensate for ischemia in the brain (1, 3, 4). Cerebral ischemia and intracranial bleeding are the main hazards of MMD (2, 5). Studies have shown that regular imaging examination in patients with unilateral MMD can significantly reduce the burden of stroke and improve the clinical prognosis (2, 6).

Digital subtraction angiography (DSA) is the traditional gold standard for pre-operative diagnosis and post-operative prognostic evaluation of MMD, nevertheless, there is concern regarding the invasiveness, high cost of the procedure and the associated exposure to radiation. In situations when DSA is not readily available, magnetic resonance angiography (MRA) combined with magnetic resonance imaging (MRI) is considered as an alternative tool (7), but MRA combined with MRI is expensive, time-intensive and the process is complicated. None of these methods are suitable for the long-term and dynamic monitoring of MMD. Ultrasound is a non-invasive, convenient, and economical method, that has been used in the pre-operative examination and post-operative prognostic evaluation of MMD (8–10). The hemodynamic parameters of extracranial internal carotid artery (EICA), PCA, STA, MA can be easily detected by ultrasound. However, there is a lack of systematic research on the association between ultrasound parameters and stroke in patients with MMD. Therefore, in this study, we used ultrasound to detect the hemodynamic parameters of the EICA, STA, MA, and PCA to identify ipsilateral stroke in patients with MMD.

## Materials and Methods

### Patient Data

We prospectively enrolled consecutive patients diagnosed with MMD based on the guidelines for MMD (7) between September 2019 and July 2020 at the Beijing Tiantan Hospital. The exclusion criteria for our study were: (1) patients who refused ultrasound examination; (2) patients with poor temporal acoustic bone window; (3) MMD patients with diseases that affect cardiac output; (4) patients who underwent prior bypass surgery; (5) patients lack of DSA, CT or MRI examination, or the interval between these examinations and ultrasound was more than 1 month, or during this period, patients exhibited new symptoms or clinical manifestations of sudden aggravation. From September 2019 to February 2020 and March 2020 to July 2020 eligible patients were assigned to training and validation datasets, respectively.

Informed consents were obtained from all eligible patients (or their parents or legal guardians for children under 18 years of age), and this study was approved by the Ethics Committee of Beijing Tiantan Hospital, Capital Medical University.

### Clinical Data

Clinical information, including age, sex, clinical manifestations, radiological findings, history of hypertension, diabetes, hyperlipidemia, smoking, and drinking were recorded. Based on the clinical manifestations on admission, the 92 hemispheres from 52 patients were diagnosed as stroke or non-stroke by the following methods: (1) stroke was defined as patients who had already experienced ischemic stroke or hemorrhagic stroke. Ischemic stroke was a new symptomatic neurological deterioration that was confirmed by MRI as cerebral infarction and could not be attributed to non-ischemic causes. Hemorrhagic stroke was defined as blood permeated into the brain parenchyma that was confirmed by CT (11). (2) Non-stroke was defined as patients who had demonstrated no signs of cerebral infarction or hemorrhage. All the clinical manifestations were confirmed by a neurosurgeon with over 5 years of experience.

### Radiological Evaluations

The criteria of collateral circulation were defined by Liu et al. (12). Anterior collateral circulation was assessed based on Suzuki stage (1, 2), Suzuki stage I to VI corresponded to scores of 6 to 1, respectively. Six points corresponded to stage I: narrowing of the internal carotid artery (ICA) apex; 5 points corresponded to stage II: initiation of moyamoya collaterals; 4 points corresponded to stage III: progressive stenosis of the ICA with intensification of moyamoya collaterals; 3 points corresponded to stage IV: development of ECA collaterals; 2 points corresponded to stage V: reduction of moyamoya vessels and intensification of ECA collaterals; and 1 point corresponded to stage VI: complete occlusion of the ICA and disappearance of moyamoya collaterals.

Posterior collateral circulation was assessed according to the anatomical extent of pial collateral blood from the PCA territory to the middle cerebral artery (MCA) and anterior cerebral artery (ACA) territory (12). Scoring of leptomeningeal collateral networks from the PCA to ACA territory was as following: (1) 1 point: blood supply to the cortical border area between the ACA and PCA territory; and (2) 2 points: blood supply over the central sulcus. Scoring of leptomeningeal collateral networks from the PCA to MCA territory was as following: (1) 1 point: the parieto-occipital branch or the anterior temporal branch of the PCA anastomoses to the MCA; (2) 2 points: blood supply extended to the sylvian fissure; and (3) 3 points: blood supply extended to the occlusion (M1 or proximal M2 segments of the MCA). A score of 0 point was assigned if there were no leptomeningeal anastomoses.

The grading score of the collateral circulation was the sum of the above anterior and posterior collateral circulation scores, it was graded as follows: grade I, 0 to 4 points; grade II, 5 to 8 points; and grade III, 9 to12 points.

### Ultrasound Examination

Ultrasound parameters were measured by 2 independent experienced sonographers (SZ and YL), blinded to the radiographic findings and clinical data. Ultrasound examination was performed using a sonographic scanner (EPIQ 7, Philips Medical Systems, Bothell, WA).

#### Extracranial Internal Carotid Artery

Carotid ultrasound was performed using a 3–12 MHz linear array probe. The patients assumed a supine position with the head slightly turned to the opposite side. The examiner adjusted the depth, grayscale, and focus according to the condition of each patient. The diameter (D) of the EICA was measured at 1–2 cm above the carotid sinus. Then color and pulse doppler were used to measure the time-averaged mean velocity (TAMV). The gain, pulse-repetition frequency, and wall filter were corrected to appropriate condition, the doppler sample size was adjusted to the same width as the vessel, the doppler angle was <60°, and after at least three cardiac cycles, the TAMV was measured. The flow volume (FV) was obtained according to the formula FV = TAMV × [(D/2)^2^ × π] (13, 14). The level of the FV in EICA was categorized as “<50 ml/min,” “50–99 ml/min,” “100–149 ml/min,” “150–199 ml/min,” or “>200 ml/min.”

#### Superficial Temporal Artery

The STA was examined using a 3–12 MHz linear array probe. The patient assumed a supine position with the head turned to the opposite side. The probe was placed before the ear at the trunk of the STA. The examiner adjusted the depth, grayscale, and focus according to the condition of each patient. Color and pulse doppler were subsequently used to measure the peak systolic velocity (PSV) of the STA. The gain, pulse-repetition frequency, and wall filter were corrected to appropriate condition, the doppler sample size was adjusted to about 1/3 of the STA, and the doppler angle was <60°. Then, the PSV of the STA was measured. The level of the PSV in STA was categorized as “ <50 cm/s” or “50–99 cm/s.”

#### Maxillary Artery

The MA was examined with a 1–5 MHz convex transducer. The patient assumed a supine position with the head turned to the opposite side. The probe was placed at the mandibular angle and pointed toward the tip of the nose. The examiner adjusted the depth, grayscale, and focus according to the condition of each patient. Color and pulse doppler were subsequently used to measure the PSV of the MA. The gain, pulse-repetition frequency, and wall filter were corrected to appropriate condition, the doppler sample size was adjusted to about 1/3 of the MA, and the doppler angle was <60°. Then, the PSV of MA was measured. The level of the PSV in MA was categorized as “<50 cm/s,” “50–99 cm/s,” or “100–149 cm/s.”

#### Posterior Cerebral Artery

Transcranial color-coded sonography (TCCS) was performed using a 1.5–3.0 MHz phased array probe. The P2 segment of the PCA was detected through the transtemporal window. Color and pulse doppler were used to measure the PSV of the PCA. The patient assumed a lateral position. The examiner adjusted the parameters of the sonographic scanner according to the condition of each patient. The depth of insonation was adjusted to 60–70 mm, the doppler sample size was 3–5 mm, the doppler angle was <60°. Then, the PSV of the PCA was measured. The level of the PSV in PCA was categorized as “<50 cm/s,” “50–99 cm/s,” “100–149 cm/s,” “150–199 cm/s,” or “>200cm/s.”

### Statistical Analysis

Statistical analyses were performed using SPSS version 24.0 (IBM Corporation, Armonk, NY). Continuous variables were described as medians (interquartile ranges), ranked variables and categorical variables were described as percentages. The Mann-Whitney *U*-test was used for continuous variables and ranked variables; the chi-square test was used for categorical variables. Weighted kappa (κ) was used to assess the interrater reliability of ultrasound parameters between the 2 sonographers in the training dataset. As for ultrasound parameters we used the parameters measured by the first rater. Multivariate logistic regression analysis was applied to identify ultrasound parameters associated with previous history of ipsilateral stroke in patients with MMD. The rms package for R software 3.6.1 was used to establish a nomogram incorporating the independent associated parameters. Then, a validation dataset was used for verification. The discrimination ability of the nomogram was evaluated using the area under the receiver operating characteristic (AUROC) curves. The consistency between the observed and the assessed probability of stroke was evaluated by calibration curve. The Hosmer-Lemeshow-test was performed to verify the suitability of the nomogram (15, 16). Meanwhile, decision curve analysis was performed to evaluate the clinical applicability of the model. All calculated *P*-values were 2-tailed, and a *P*-value <0.05 was considered statistically significant.

## Results

### Patient Characteristics

During the study period, of 205 patients with MMD, 13 patients refused ultrasound examination, 8 patients with poor temporal acoustic bone window, 10 patients with diseases that affect cardiac output, 48 patients underwent prior bypass surgery and 74 patients lack of DSA, CT, or MRI examination, or the interval between these examinations and ultrasound was more than 1 month, or during this period, patients exhibited new symptoms or clinical manifestations of sudden aggravation were excluded. Finally, 52 patients (92 hemispheres) met the inclusion criteria were included in the analysis. Among them, from September 2019 to February 2020, 36 patients (65 hemispheres) were assigned to the training dataset, from March 2020 to July 2020, 16 patients (27 hemispheres) were assigned to the validation dataset. Of 52 eligible patients, 12 patients had unilateral hemispheric involvement and 40 patients had bilateral hemispheric involvement. The demographics, clinical history, angiographic findings and ultrasound parameters of patients with MMD in the training and validation datasets were shown in Table 1. Stroke accounted for 38.5% (25/65) in the training dataset, and 40.7% (11/27) in the validation dataset. There were no differences in the above data between the two datasets.

**Table 1:** Baseline characteristics of eligible patients.

**Characteristics**	**Training dataset (*n* = 65 hemispheres)**	**Validation dataset (*n* = 27 hemispheres)**	***P*-value**
Age, years	38.0 (28.5–46.0)	37.0 (34.0–45.0)	0.725
Sex, male, *n* (%)	30 (46.2)	12 (44.4)	0.881
Clinical history, *n* (%)			
Hypertension	15 (23.1)	6 (22.2)	0.929
Diabetes	4 (6.2)	2 (7.4)	0.825
Hyperlipidemia	5(7.7)	1 (3.7)	0.809
Smoking	5 (7.7)	2 (7.4)	0.508
Drinking	5 (7.7)	2 (7.4)	0.508
Stroke, *n* (%)	25 (38.5)	11 (40.7)	0.838
Suzuki stage, *n* (%)			0.877
I	5 (7.7)	3 (11.1)	
II	15 (23.1)	6 (22.2)	
III	21 (32.3)	8 (29.6)	
IV	12 (18.5)	5 (18.5)	
V	11 (16.9)	4 (14.8)	
VI	1 (1.5)	1 (3.7)	
Collateral circulation, *n* (%)			0.241
Grade I	13 (20)	3 (11.1)	
Grade II	44 (67.7)	19 (70.4)	
Grade III	8 (12.3)	5 (18.5)	
Ultrasound parameters			
EICA_FV_ (ml/min)			
<50	15 (23.1)	6 (22.2)	0.772
50–99	15 (23.1)	6 (22.2)	
100–149	14 (21.5)	9 (33.3)	
150–199	12 (18.5)	3 (11.1)	
>200	9 (13.8)	3 (11.1)	
PCA_PSV_ (cm/s)			0.862
<50	7 (10.8)	1 (3.7)	
50–99	19 (29.2)	9 (33.3)	
100–149	22 (33.8)	11 (40.7)	
150–199	11 (16.9)	4 (14.8)	
>200	6 (9.2)	2 (7.4)	
STA_PSV_ (cm/s)			0.871
<50	13 (20)	5 (18.5)	
50–99	52 (80)	22 (81.4)	
MA_PSV_ (cm/s)			0.359
<50	17 (26.1)	6 (22.2)	
50–99	42 (64.6)	16 (59.3)	
100–149	6 (9.2)	5 (18.5)	

*EICA_FV_, flow volume of extracranial internal carotid artery; PCA_PSV_, peak systolic velocity of posterior cerebral artery; STA_PSV_, peak systolic velocity of superficial temporal artery; MA_PSV_, peak systolic velocity of maxillary artery*.

### Interrater Reliability of Ultrasound Parameters Between 2 Sonographers

The interrater reliability between 2 sonographers was good for the FV of the EICA (κ: 0.79, 95 % CI: 0.70–0.89) and the PSV of the MA (k: 0.76, 95 % CI: 0.62–0.91); The interrater reliability was excellent for the PSV of the STA (k: 0.82, 95 % CI: 0.65–0.99) and the PSV of the PCA (k: 0.83, 95 % CI: 0.74–0.93).

### Multivariate Logistic Regression Analysis of Associated Ultrasound Parameters for Previous History of Ipsilateral Stroke in Patients With MMD

Univariate analysis revealed that the FV of the EICA and the PSV of the PCA were associated ultrasound parameters for previous history of ipsilateral stroke in patients with MMD. Multivariate analysis revealed that when the variables were not adjusted in model 1, the FV of the EICA (OR = 0.511, 95% CI: 0.319–0.821, *P* = 0.005) and the PSV of the PCA (OR = 0.550, 95% CI:0.313–0.966, *P* = 0.037) were independent associated ultrasound parameters for stroke. After adjusting for age and sex in model 2, the FV of the EICA (OR = 0.476, 95% CI: 0.282–0.804, *P* = 0.005) and the PSV of the PCA (OR = 0.545, 95% CI: 0.311–0.955, *P* = 0.034) were still independent associated ultrasound parameters for stroke (Table 2). By using these ultrasound parameters, we constructed a model to identify stroke in patients with MMD.

**Table 2:** Logistic regression for ultrasound parameters to identify stroke in patients with MMD.

**Variables**	**Univariable analysis**		**Multivariable analysis**			
			**Model 1^a^**		**Model 2^b^**	
	**OR (95% CI)**	***P***	**OR (95% CI)**	***P***	**OR (95% CI)**	***P***
Age, years	1.007 (0.973–1.042)	0.691				
Male sex	0.960 (0.351–2.626)	0.937				
**History of risk factors**						
Hypertension	1.088 (0.334–3.541)	0.889				
Diabetes	1.652 (0.218–12.545)	0.627				
Hyperlipidemia	1.072 (0.166–6.912)	0.941				
Smoking	2.591 (0.401–16.721)	0.317				
Drinking	2.591 (0.401–16.721)	0.317				
**Ultrasound parameters**						
EICA_FV_	0.495 (0.314–0.779)	0.002	0.511 (0.319–0.821)	0.005	0.476 (0.282–0.804)	0.005
STA_PSV_	0.672 (0.197–2.293)	0.525				
MA_PSV_	0.857 (0.356–2.064)	0.731				
PCA_PSV_	0.520 (0.307–0.879)	0.015	0.550 (0.313–0.966)	0.037	0.545 (0.311–0.955)	0.034

*OR, odds ratio; 95% CI, 95% confidence interval; EICA_FV_, flow volume of extracranial internal carotid artery; PCA_PSV_, peak systolic velocity of posterior cerebral artery; STA_PSV_, peak systolic velocity of superficial temporal artery; MA_PSV_, peak systolic velocity of maxillary artery*.

a *Unadjusted*.

b *Adjusted for age and sex*.

### Construction of the Nomogram

**Figure 1:**
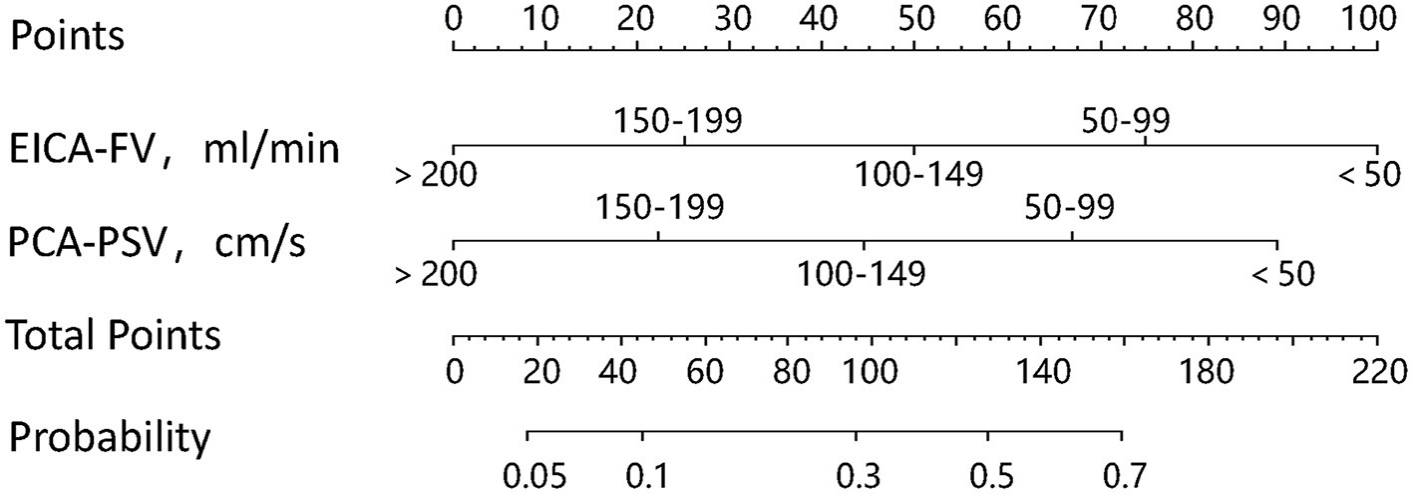
The nomogram used to identify stroke in patients with MMD. To use the nomogram, first, find the value of each variable, draw a vertical line to the points axis to find the corresponding points, add the points of each variable to get the total points, and then, draw a vertical line from the total points axis to find the probability of stroke.

**Figure 2:**
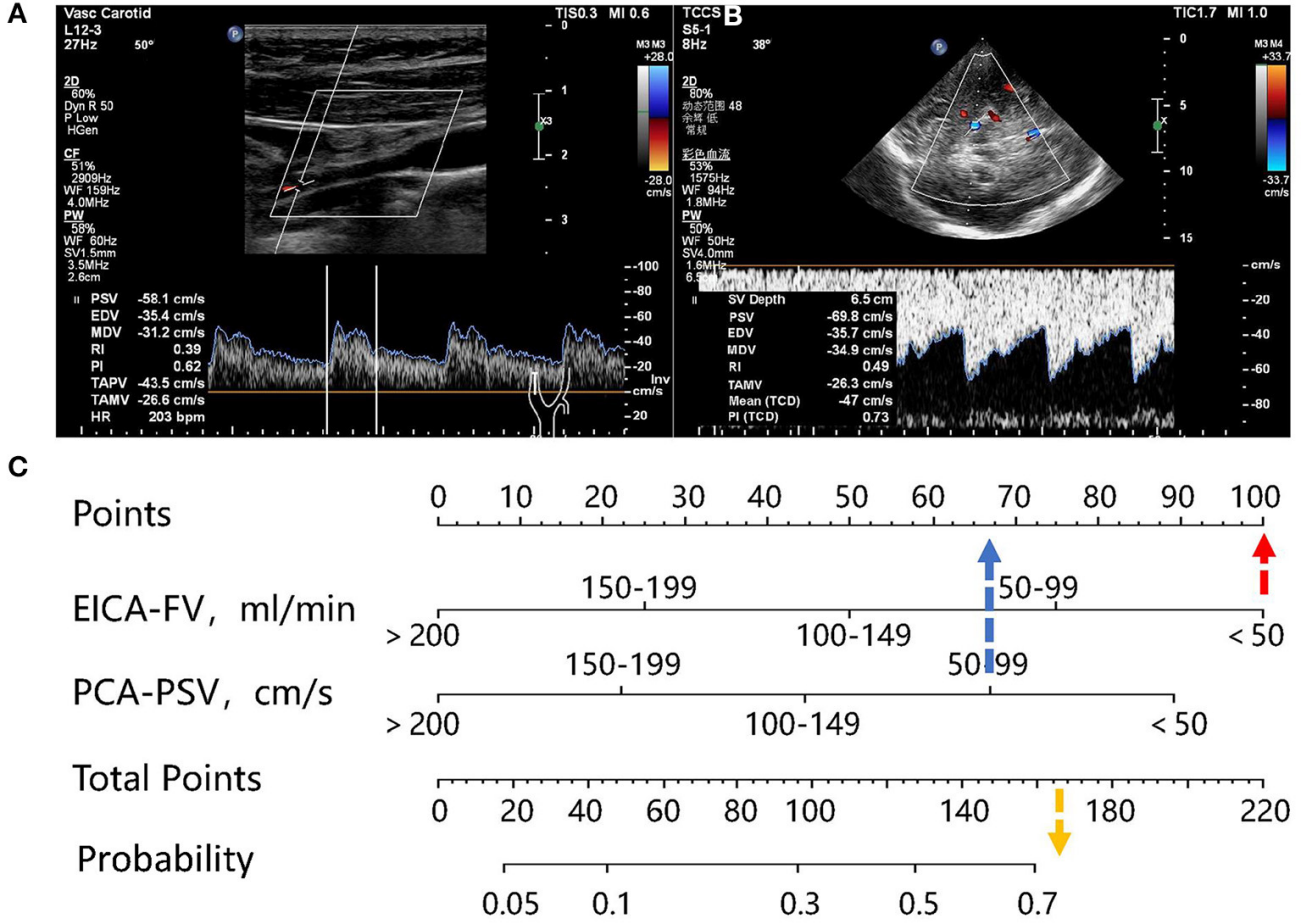
Representative case. **(A)** A 33-year-old woman with MMD. Carotid ultrasonography showed that the diameter of right EICA was 0.19 cm, the TAMV was 21.2 cm/s, and the FV was 36.1 ml/min. **(B)** The TCCS showed that the PSV of the PCA was 69.8 cm/s. **(C)** the FV of the right EICA was 36.1 ml/min, corresponding to100 points, the PSV of right PCA was 69.8 cm/s, corresponding to 67 points, and the sum was 167 points, which can be converted to a probability of >70%.

A nomogram was constructed based on the independent associated ultrasound parameters identified by the multivariable analysis to differentiate between stroke and non-stroke patients with MMD (Figure 1). With the nomogram, the identification ability could be calculated by summing the points of each variable, and then locating the sum on the total points axis. In a representative case, a 33-year-old woman with MMD, the FV of the right EICA was 36.1 ml/min (100 points), the PSV of the right PCA was 69.8 cm/s (67 points), and the sum was 167 points, which could be converted into >70% probability of right hemisphere stroke (Figure 2).

### Performance of the Nomogram

**Figure 3:**
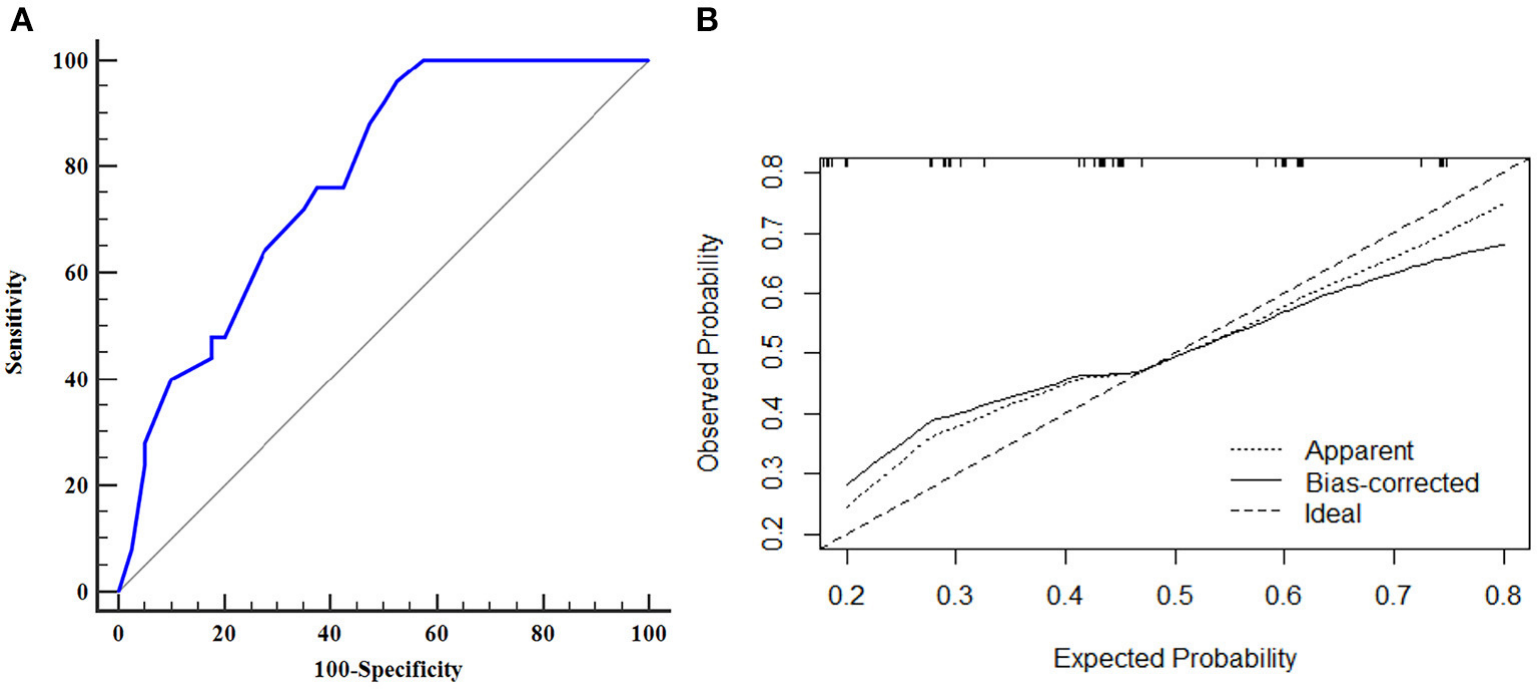
ROC curve and calibration plot of the nomogram in the training dataset. **(A)** ROC curve of the nomogram, AUROC was 0.776 (95% CI, 0.656–0.870); **(B)** Calibration plot of the nomogram in the training dataset.

**Figure 4:**
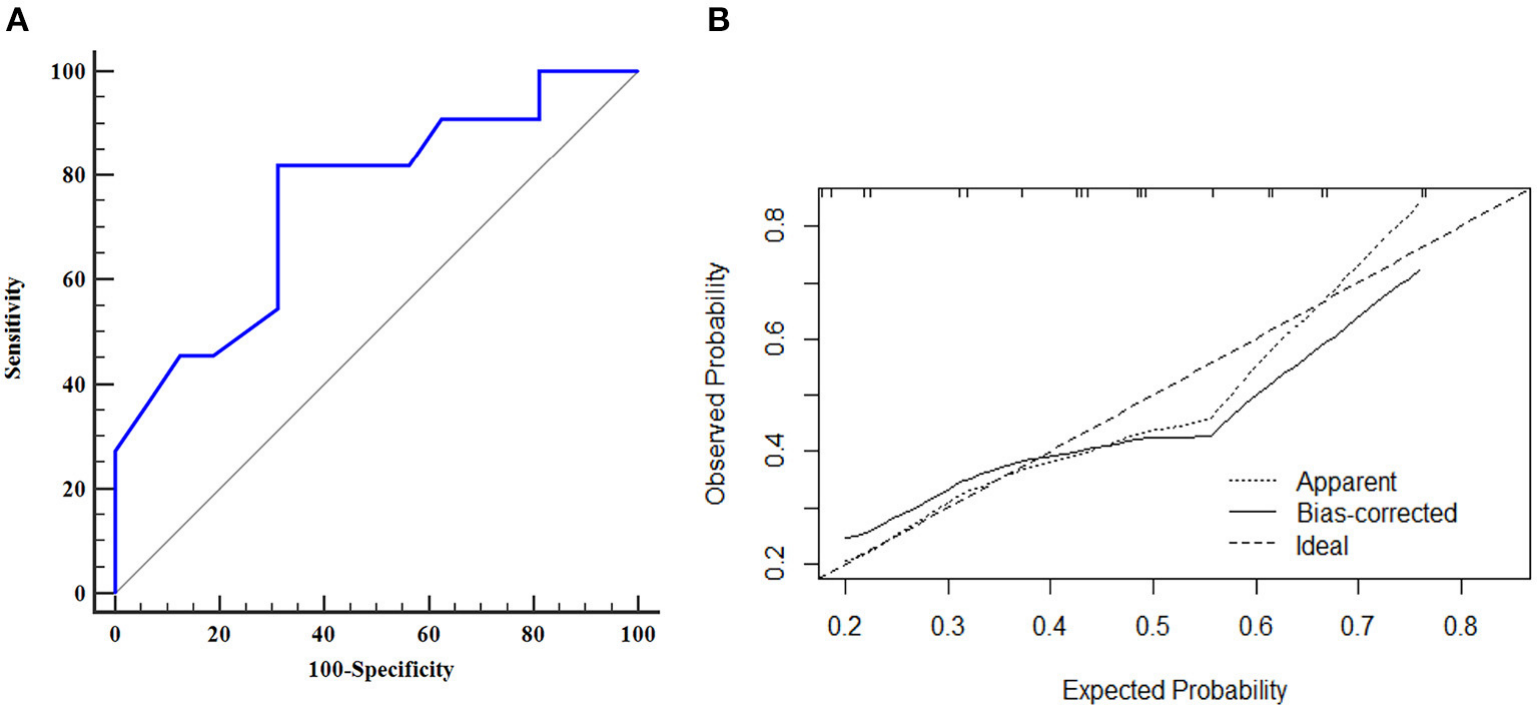
ROC curve and calibration plot of the nomogram in the validation dataset. **(A)** ROC curve of the nomogram, AUROC was 0.753 (95% CI, 0.550–0.897); **(B)** Calibration plot of the nomogram in the validation dataset.

**Figure 5:**
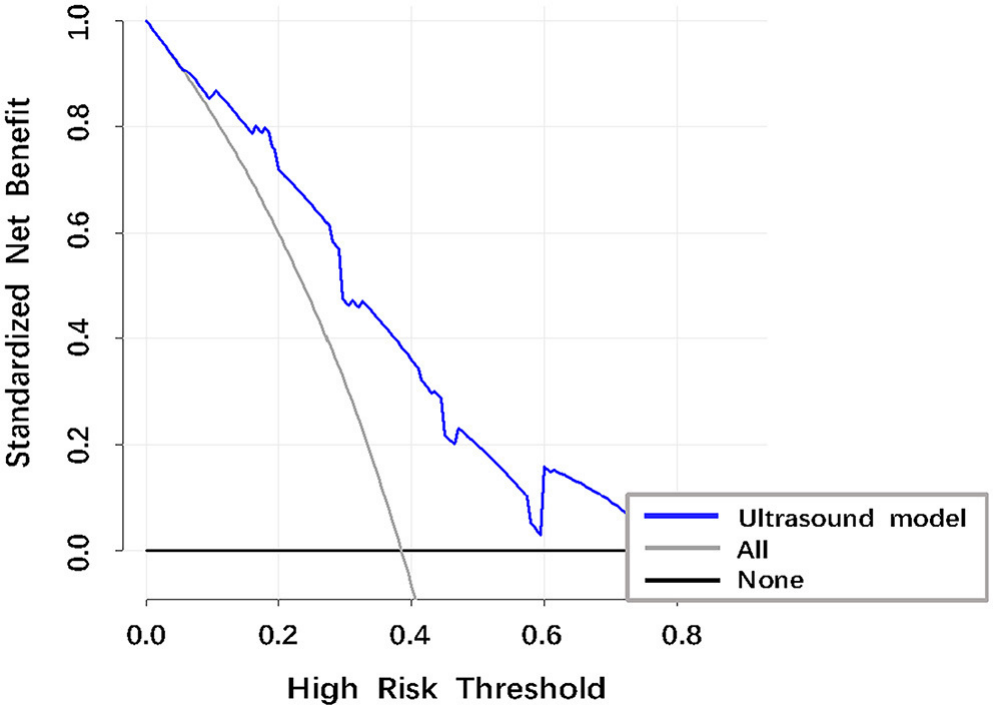
Decision curve analysis (DCA) of the ultrasound model to identify stroke in patients with MMD. The vertical axis was the standardized net benefit. The horizontal axis was the corresponding risk threshold. The DCA showed that if the threshold probability ranged from 0.10 to 0.75, using the nomogram to identify stroke in patients provided a net benefit.

The AUROC curves was 0.776 (95% CI, 0.656–0.870) in the training dataset and 0.753 (95% CI, 0.550–0.897) in the validation dataset suggested that the model had good discrimination ability (Figures 3A, 4A). The *P*-value of the Hosmer-Lemeshow test was 0.311 and 0.296 in the training and validation datasets, respectively, indicating a good fit of the model. The calibration curve of the nomogram showed good consistency between the observed and assessed outcomes in both the training and validation datasets (Figures 3B, 4B). The decision curve analysis (DCA) indicated that when the threshold probability ranged from 0.1 to 0.75, clinicians or patients could obtain a net benefit by using this nomogram to differentiate between stroke and non-stroke patients with MMD, and that the nomogram was therefore clinically useful (Figure 5).

## Discussion

In this study, we constructed an easy-to-use nomogram incorporating the FV of the EICA and the PSV of the PCA to identify previous history of ipsilateral stroke in patients with MMD. The discrimination ability of the nomogram was evaluated by using the AUROC, with an AUROC of 0.5 being defined as meaningless; 0.5–0.7 being defined as fair; 0.7–0.9 being defined as good; and >0.9 being defined as excellent. Our nomogram showed good discrimination and good calibration in both the training and validation datasets. The DCA indicated that the nomogram was clinically useful.

The clinical manifestations and outcomes of patients with MMD are heterogeneous. Digital subtraction angiography (DSA) is the traditional gold standard for pre-operative diagnosis and post-operative prognostic evaluation of MMD. According to DSA findings, the severity of the disease is often divided into six progressive stages, which is called as Suzuki stage and were defined by Suzuki and Takaku in 1969 (1). However, DSA is invasive and requires exogenous contrast agent and ionizing radiation. Although it has improved our understanding of MMD, it is not an ideal method for long-term monitoring and assessing risk of poor clinical outcomes in patients with MMD.

Ultrasound is a non-invasive, repeatable, and economical technique, that has been used to screen MMD, detect moyamoya spontaneous anastomosis pre-operatively and evaluate the prognosis post-operatively (8–10). Hong et al. used ultrasound to explore the FV of the EICA in MMD patients, the results showed that the FV of the EICA is inversely correlated with Suzuki stage (17). Yasuda evaluated the ratio of the diameter of the EICA to that of the common carotid artery by using carotid ultrasound and cerebral angiography, and the results confirmed that the lower the ratio, the more likely a patient is to experience ipsilateral cerebral vascular events (18). Our study coincided with previous studies. We found that the FV of the EICA is an independent associated parameter for previous history of ipsilateral stroke in patients with MMD. The FV of the EICA is the product of the cross-sectional area and the time-averaged mean velocity (13, 14). As MMD progresses, the diameter of the EICA gradually decreases, which is known as the bottle neck sign (18, 19), leading to a decrease in the cross-sectional area of the EICA. As stenosis of the terminal portions of the ICA progresses, the increased vascular resistance at the distal portions leads to decreased velocity of the EICA, which eventually results in a reduction in the FV of the EICA. Due to the reduced FV of the EICA, the ACA and MCA territory are susceptible to hypoperfusion (20). Hypoperfusion increases the susceptibility to ischemia, abnormal hemodynamics can lead to ischemic stroke (13). Cerebral hemorrhage is an adverse consequence of a compensatory response to cerebral ischemia, rupture of the fragile moyamoya vessels and Willis aneurysm under abnormally increased hemodynamic stress can result in hemorrhagic stroke (21). Therefore, the FV of the EICA decreased as Suzuki stage advanced, the FV of the EICA is an independent associated parameter for previous history of ipsilateral stroke in patients with MMD.

However, in some cases, the Suzuki stage may not correlate with clinical severity, because the main blood vessels of anterior circulation occlusions may be compensated by collateral vessels. Leptomeningeal collaterals from the PCA are regarded as the main collateral vessels, and transdural collaterals from the ECA can also compensate for ischemia in the brain (1, 3, 22, 23). Previous studies have shown that the more extensive the collateral vessels produced by the PCA supply the ACA and MCA territory, the less likely a patient is to suffer from ischemic stroke and parenchymal hemorrhage (12, 24, 25). Our research seems to be consistent with previous studies. In our study, TCCS was used to detect the PSV of the PCA. Although TCCS cannot directly and stereoscopically display the lumen structure of intracranial vessels or the density of abnormal moyamoya vessels, but it can reflect the compensation from the PCA to anterior circulation by measuring the flow velocity. We found that the PSV of the PCA is an independent associated parameter for previous history of ipsilateral stroke in patients with MMD. In our study, we chose the P2 segment of the PCA to explore hemodynamic changes, because in some cases, the PCA is partially or completely derived from the ICA, with the P1 segment being absent or exhibiting dysplasia. The P1 segment of the PCA mainly supplies the thalamus and basal ganglia. In addition, stenosis and occlusion of the PCA initially occurs in its proximal segment. Therefore, study of the P2 segment is more meaningful (26, 27). We found that the higher the PSV of the PCA, the less likely a patient is to experience a stroke in the ipsilateral hemisphere. The cause of this is that a high PSV of the PCA can lead to abundant collateral circulation. If the P1 segment of the PCA is narrowed, the P2 segment presents with low-velocity blood flow, it cannot form abundant collateral circulation. Therefore, the higher the PSV of PCA, the more collateral circulation formed by the PCA, resulting in lower stroke occurrence in patients with MMD (27). We also measured the PSV of the STA and MA in patients with MMD, However, these parameters were not found to be statistically significant in the univariate and multivariate analyses.

Recently, Liu et al. proposed a new MMD grading system to assess clinical symptoms by combining Suzuki stage with the leptomeningeal system from the PCA. This new MMD grading system correlates well with hemodynamic status and clinical symptoms, and contributes to risk stratification and prognostic predictions in patients with MMD (13). In our study, we constructed a nomogram to identify stroke in patients with MMD incorporating ultrasound parameters of the PCA and EICA, which has good discrimination and calibration. These findings indicated that the nomogram might become an important method for identification of stroke in patients with MMD, clinicians might take targeted individual preventive and control measures.

Our study has some limitations. First, because the incidence of MMD is low, our sample size was relatively small. We divided the patients into stroke group and non-stroke group according to their clinical manifestations, but did not further divide the patients into ischemic stroke group, hemorrhagic stroke group, transient ischemic attack group, and asymptomatic group. Second, this study was a single-center study, and there was no ultrasound examination before stroke onset in patients with MMD. Therefore, Further validation with prospective registration and long-term follow-up is needed to prove the predictive ability of our findings.

## Conclusions

Ultrasound parameters of EICA and PCA are associated with previous history of ipsilateral stroke in patients with MMD. The present ultrasound-based nomogram may provide information to identify MMD patients with high risk of stroke, large-scale and prospective longitudinal cohort studies are needed to validate its predictability.

## Data Availability Statement

The raw data supporting the conclusions of this article will be made available by the authors, without undue reservation.

## Ethics Statement

The studies involving human participants were reviewed and approved by Institutional Review Board of Beijing Tiantan Hospital, Capital Medical University, Beijing, China. Written informed consent to participate in this study was provided by the participants' legal guardian/next of kin.

## Author Contributions

SZ, PG, YL, and JW: conception and design. SZ, PG, YL, JW, ZS, JZ, and LH: acquisition of data. SZ and PG: analysis and interpretation of data. SZ: drafting the article. WH: approved the final version of the manuscript on behalf of all authors. LC, DZ, and WH: study supervision. All authors critically revising the article and reviewed submitted version of manuscript.

## Conflict of Interest

The authors declare that the research was conducted in the absence of any commercial or financial relationships that could be construed as a potential conflict of interest.
